# Evaluation of the Working Mechanism of a Newly Developed Powered Ankle–Foot Orthosis

**DOI:** 10.3390/s24206562

**Published:** 2024-10-11

**Authors:** Laure Everaert, Roy Sevit, Tijl Dewit, Koen Janssens, Jolien Vanloocke, Anja Van Campenhout, Luc Labey, Luiza Muraru, Kaat Desloovere

**Affiliations:** 1 Department of Rehabilitation Sciences, Faculty of Movement and Rehabilitation Sciences, KU Leuven, 3001 Leuven, Belgium; laure.everaert@kuleuven.be (L.E.); tijl.dewit@kuleuven.be (T.D.); jolien.vanloocke@kuleuven.be (J.V.); 2 Centre of Expertise Care and Well-Being, Research Group Mobilab & Care, Thomas More University of Applied Sciences, Campus Geel, 2440 Geel, Belgium; roy.sevit@thomasmore.be (R.S.); koen.janssens@thomasmore.be (K.J.); luiza.muraru@thomasmore.be (L.M.); 3 Clinical Motion Analysis Laboratory, University Hospitals Leuven, 3000 Leuven, Belgium; 4 Department of Development and Regeneration, Faculty of Medicine, KU Leuven, 3001 Leuven, Belgium; anja.vancampenhout@uzleuven.be; 5 Pediatric Orthopedics, Department of Orthopedics, University Hospitals Leuven, 3001 Leuven, Belgium; 6 Department of Mechanical Engineering, Faculty of Engineering Technology, KU Leuven, 3001 Leuven, Belgium; luc.labey@kuleuven.be

**Keywords:** cerebral palsy, ankle–foot orthosis, exoskeleton, biomechanics, gait

## Abstract

Ankle–foot orthoses (AFOs) are commonly prescribed to children with cerebral palsy (CP). The conventional AFO successfully controls the first and second ankle rocker, but it fails to correct the third ankle rocker, which negatively effects push-off power. The current study evaluated a new powered AFO (PAFO) design, developed to address the shortcomings of the conventional AFO. Eight children with spastic CP (12.4 ± 3.4 years; GMFCS I-III; 4/4-♂/♀; 3/5-bi/unilateral) were included. Sagittal kinematic and kinetic data were collected from 20 steps during barefoot walking, with conventional AFOs and PAFOs. In the PAFO-condition, an actuation unit was attached to a hinged AFO and through push–pull cables to a backpack that was carried by the child and provided patient-specific assistance-as-needed. SnPM-analysis indicated gait cycle sections that differed significantly between conditions. For the total group, differences between the three conditions were found in ankle kinematics (49.6–66.1%, *p* = 0.006; 88.0–100%, *p* = 0.011) and angular velocity (0.0–6.0%, *p* = 0.001; 45.1–51.1%, *p* = 0.006; 62.2–73.0%, *p* = 0.001; 81.2–93.0%, *p* = 0.001). Individual SnPM-analysis revealed a greater number of significant gait cycle sections for kinematics and kinetics of the ankle, knee, and hip. These individual results were heterogeneous and specific per gait pattern. In conclusion, the new PAFO improved the ankle range-of-motion, angular velocity, and power during push-off in comparison to the conventional AFO.

## 1. Introduction

Cerebral palsy (CP) is the world’s leading cause of permanent motor disabilities in childhood, with a prevalence of 1.48 per 1000 live births. [1]. Primary problems such as abnormal muscle tone and decreased strength and selectivity, as well as subsequent contractures and deformities, often result in pathological gait patterns. Deficiencies in ankle control are common in individuals with CP and contribute significantly to gait abnormalities [2]. Improving disturbed gait is an important treatment goal, since it is associated with functional independence, physical activity levels, and participation in society [3,4]. It is common practice to prescribe ankle–foot orthoses (AFOs) for ambulatory children with CP to improve their gait, prevent secondary deformities, provide an improved base of support, and/or compensate for muscle weakness [5]. Most prescribed AFOs effectively control the first and, to some extent, the second ankle rocker, as well as ankle motion during the swing phase. However, the AFO has a negative impact on power generation during the third ankle rocker (i.e., push-off), due to the introduced limited ankle plantar flexion motion [6]. The effect of the AFO on gait was found to be different for specific barefoot gait patterns (i.e., dropfoot; genu recurvatum; jump gait; apparent equinus; crouch gait) [6,7].

Although most ambulatory children with CP are prescribed AFOs, their designs have seen minimal significant advancement over the past decades [8]. In contrast, powered orthoses/exoskeletons represent an emerging research field that aims to address some of the inherent limitations of conventional orthoses. A powered ankle–foot orthosis (PAFO) is an ankle exoskeleton that aids to achieve a more normal ankle motion, including improved ankle power generation, which cannot be achieved by conventional AFOs. The working mechanism involves provision of torques, whereby the ankle torque is measured in real time and additional torque is externally applied, which is timed to the patient’s demands [9]. So far, a few PAFO designs have been developed for children with CP [8,10,11,12]. Previously reported improvements of these exoskeletons include a reduced metabolic cost of walking, increased walking speed and increased knee and hip extension during stance [8,10,11,12,13]. However, the results of these previous studies must be interpreted with caution, due to the small sample sizes and varying methodological quality [6]. As outlined in the review of Hunt et al. [14], several factors may be responsible for the overall outcome of any exoskeleton model. Specifically, the influence of adaptation time, torque conditions, timing of assistance, the joint at which assistance is provided, the weight of the device, and the time points at which data are collected is significant. Existing PAFOs are all in alpha stages and developed as a one-size-fits-all. However, it is critical that orthoses are patient-tailored in order to provide the best comfortable fit, considering that pressure points and subsequent skin-damage are the main reason for intolerance of AFOs. Furthermore, previous publications have mainly focused on studying the effect of the PAFO on spatio-temporal parameters, such as cadence and walking speed, and on predefined discrete gait parameters that are extracted from the gait continuous waveforms, such as overall gait deviation indexes, ankle range of motion (ROM), peak knee and hip extension/flexion during stance, peak ankle moment, and power during stance [14]. However, it has been shown that AFOs may impact several or sometimes all sections of the continuous kinematic and/or kinetic gait waveforms [6]. Therefore, it is considered relevant to also investigate the effect of the PAFO on the entire gait cycle.

The aim of the current pilot study was to examine whether a newly designed PAFO with patient-specific fine-tuning (i.e., assistance-as-needed) could improve the gait pattern of children with CP, compared to their conventional AFOs. We first investigated the effect of the PAFO on predefined discrete gait parameters and on the entire gait waveforms, for a total CP-group. Because of the above-highlighted gait-pattern-specific effect of AFOs, we also described the PAFO effect for the individual patients who were classified per barefoot gait pattern. Based on previous literature findings [14] and on the working mechanism of the PAFO, we a priori expected that the PAFO would (a) increase ankle ROM, angular velocity, and power during the push-off phase, and (b) improve the proximal joint kinematics (i.e., reduce knee and hip flexion for crouch and apparent equinus and reduce knee hyperextension for genu recurvatum).

## 2. Materials and Methods

### 2.1. Mechanical Design

**Figure 1:**
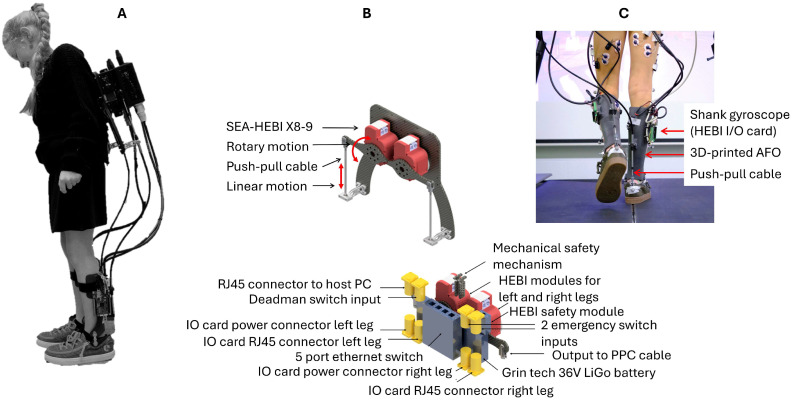
Overview of (**A**) the ankle exoskeleton being worn by a healthy child with details on (**B**) the actuation module; and (**C**) a back view of the 3D-printed ankle–foot orthosis.

We designed a battery-powered ankle exoskeleton or PAFO providing bilateral assistance-as-needed for both plantar- and dorsiflexion movements. The PAFO consisted of an actuation and an ankle module (Figure 1). To mitigate the metabolic detriment associated with increased distal leg mass [15], the actuation module was integrated into a backpack that weighs approximately 4.3 kg. The actuation unit housed 2 off-the-shelf Series Elastic Actuators (HEBI X8-9, HEBI Robotics, Pittsburgh, PA, USA; each weigh 475 g), a LiGo battery (Grin Technologies, Vancouver, BC, Canada; 36 V–2.7 Ah or 98 Wh weighing 610 g) a 5-port ethernet switch, a HEBI POE safety module, connectors to the safety switches, and cabling. The X8-9 actuators could provide a continuous torque of 8 Nm and a peak torque of 20 Nm. The force was transmitted from the actuators to the ankle joint through push–pull cables (Fortatech AG, St. Gallen, Switzerland) which converted the rotation motion of the actuators into translation [16] and further stirred up plantar- and dorsiflexion motion of the footplates relative to the shank. The ankle module was a patient-specific three-dimensional (3D)-printed articulated AFO. The shank cuffs and foot plates were digitally designed based on corrected 3D-scan models of the legs to ensure optimal fit. The attachments for the push–pull cables were also generated during the digital design process, which resulted in the alignment of the mechanical assistance torque axis with the ankle’s anatomical rotation axis [17]. The cuffs were connected through pivot joints (Pivot^TM^, Pivot^TM^, LaunchPad Europe, Oostkamp, Belgium) which were aligned digitally with the anatomical axis and mounted on the lateral and medial sides of the AFO.

### 2.2. Control System

The PAFO could provide varying assistance depending on the phases of the gait cycle. Therefore, a gait segmentation algorithm [18] was implemented into the control loop of the ankle exoskeleton. This algorithm used angular velocity measured by two single-axis gyroscopes to detect seven phases of a standard walking gait cycle (i.e., loading response, midstance, terminal stance, preswing, initial swing, midswing and terminal swing). The gyroscopes were part of the inertial measurement units (IMUs) located in HEBI IO cards which were placed on supports in the 3D-printed shank cuffs of the AFO.

The PAFO utilized an impedance control strategy, where the external assistance complemented the movement generated by the subject. The torque delivered by the PAFO depended on the deviation from a phase-specific target angle. For each detected gait phase, a target angle and stiffness, which were also patient-specific, could be set in the controlling software. The resulting assistance applied to the ankle for each phase was based on the difference between the actual measured ankle angle and the phase-specific target angle multiplied by the phase-specific stiffness (i.e., an impedance controller). The actual ankle angle and interaction force were derived from the HEBI actuator output encoder and series elasticity deflection, respectively.

### 2.3. Participants

Eight children with CP participated in this study. The children were recruited at the CP Reference Centre at the University Hospital of Leuven (Belgium). Inclusion criteria were (a) spastic uni- or bilateral CP diagnosis, (b) age between 6 and 17 years, (c) gross motor function classification system (GMFCS) level of I, II, or III, and (d) walking daily with their AFOs prescribed by a medical team as part of their standard clinical care. Participants were excluded if they had (a) severe contractures or spasticity, making it impossible to properly wear an AFO, (b) lower cognitive capacities hindering them from understanding instructions (severe cognitive impairment), (c) previous surgery on bones and/or muscles of the legs in the 12 months prior to assessment, and (d) ataxia or dystonia.

In addition, we used a control dataset of typically developing (TD) children. This TD database was previously established at the treadmill lab of the clinical motion analysis laboratory and included the gait data from 18 TD children (9.5 y ± 3.3 y). The data of the TD children were used as a reference in the visualization of the results and for the calculation of the gait indices (i.e., gait profile score (GPS) and gait variable scores (GVSs), as explained in more detail below).

### 2.4. Study Protocol

**Figure 2:**
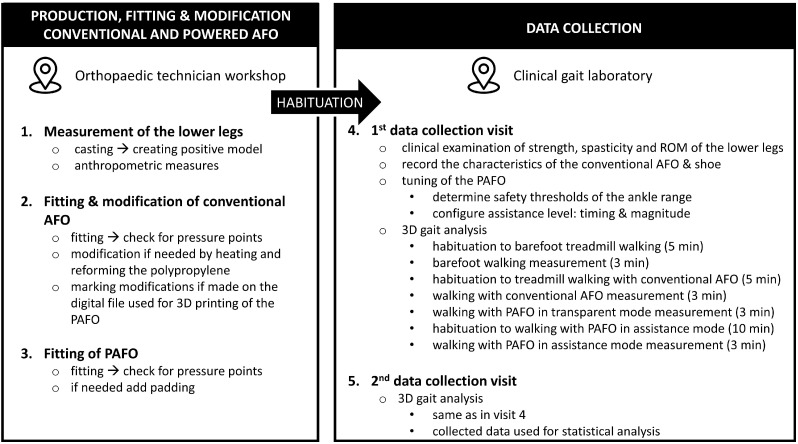
Overview of the 5 sessions and testing protocol. The habituation period between steps 1–3 and steps 4–5 ranged from 2 weeks to 1 month. Abbreviations: AFO = ankle–foot orthosis, PAFO = powered ankle–foot orthosis, min = minutes, 3D = three dimensional.

The study was executed in a span of 5 sessions per participant, requiring 3 visits for the production, fitting, and modification of the conventional AFO and PAFO and 2 measurement sessions (Figure 2).

In the months preceding the study, each participant received a clinical overground 3D gait analysis (3DGA) and a clinical examination restricted to the ankle and knee joint (i.e., measuring joint ROM and bony deformities, spasticity based on the Modified Ashworth Scale (MAS) and modified Tardieu scale, muscle strength based on manual testing, and selective muscle control based on a selectivity scale of the lower limbs) by an expert clinician at the Clinical Motion Analysis Laboratory, as part of their routine clinical follow-up [19]. The barefoot gait pattern for each participant was classified as dropfoot, equinus, genu recurvatum, true equinus, jump gait, apparent equinus, or crouch, according to the gait classification system introduced by Papageorgiou et al. (2019) [7].

In total, 3 visits were required to deliver a patient-tailored 3D-printed PAFO. More information about the production process of the PAFO is presented in [17]. The participants were fitted with the 3D-printed ankle module (without the actuation module and transmission cables) and underwent a habituation period ranging from 2 weeks to 1 month. During this period, they walked at home for 30 min daily while wearing the 3D-printed cuffs. During the habituation period, the multidisciplinary team of assessors (involving movement analysis specialists and engineers) discussed the previously collected overground clinical gait analysis data of the participant (in the barefoot overground condition and with the conventional AFO). Based on this discussion, the required assistance (i.e., magnitude and timing) was estimated.

After the habituation period, two data collection sessions (i.e., visits 4 and 5) were planned, involving a 3DGA, with 5 to 14 days between both sessions. During the two data collection sessions, gait analysis was performed in the barefoot condition, while walking with their conventional AFO and while walking with the PAFO. The conventional AFOs were always combined with their habitual flat shoes, while the PAFO was always combined with the same type of flat shoe (Billy Footwear^®^, Kent, WA, USA). During visit 4, the main goal was to optimally tune the PAFO for each participant while providing sufficient time for habituation to walking with the device (namely, until we visually observed a consistent gait, which was usually after 15–30 min). To facilitate gradual adaptation, the actuator settings were progressively increased from transparent mode to full assistance mode in 25% increments. The final settings determined during visit 4 were then used in visit 5, during which the final kinematic and kinetic data were collected and used for further analysis.

The 3DGAs were performed with 10 optoelectronic cameras (Vicon Motion Systems, Oxford, UK) in combination with an instrumented treadmill (GRAIL–Motek Medical, Houten, The Netherlands), in the treadmill gait laboratory. To prevent any fall incidences, a safety harness was worn during standing and walking on the treadmill at all times. During the barefoot treadmill habituation, participants indicated a comfortable speed, which was used as the basis for the walking speed on the treadmill. The initial pace was set 10% lower than the observed walking speed throughout the previous overground 3DGA (because CP children commonly walk around 10% slower on a treadmill [20]) and was incrementally adjusted until the most comfortable walking speed was achieved. Sixteen light-reflecting markers were fixed to the participant’s skin according to the lower body PlugInGait marker model (Oxford metrics, Oxford, UK). During the (P)AFO walking condition, the forefoot, heel, and ankle markers were repositioned on the shoe/AFO focussing on optimal joint axis alignment with the underlying anatomy.

Using the Vicon Nexus Software (version 2.12.0, Oxford Metrics, Oxford, UK), continuous gait data were segmented into different gait cycles and time-normalized to the duration of the cycle. The kinematic and kinetic waveforms were obtained by yielding 101 points of data per curve. The kinematic data included cardan angles (expressed in degrees) of the ankle, knee, and hip, decomposed in different anatomical planes, based on the lower body PlugInGait kinematic model. For this study, only the sagittal plane was considered. The kinetic data included internal joint moments and power, normalized for body weight (expressed in Nm/kg and W/kg, respectively). These were defined through inverse dynamics, based on the kinematic and force-plate data.

No adverse effects were reported, and the device was perceived as comfortable by all participants.

### 2.5. Data and Statistical Analysis

Only the data from visit 5 were used for further analysis. Data were collected from 3 different walking conditions (i.e., barefoot, with AFOs, and with PAFOs). In this study, 2 types of outcome measures were selected: discrete gait parameters and continuous gait waveforms. The discrete gait parameters consisted of 2 spatiotemporal measures (i.e., cadence and step length) and overall gait indices, including the GPS and GVS for the ankle, knee, and hip in the sagittal plane. The GPS is based upon 15 clinically important trajectories of joint angles (pelvic tilt, obliquity, rotation and hip flexion, abduction, internal rotation, knee flexion, dorsiflexion, and foot progression), which are expressed as GVSs, whereby each GVS represents the root mean square difference between a joint-specific time-normalized kinematic waveform and the mean data for typically developing children [21]. The GPS thus expresses the overall gait deviation, while the GVSs summarize overall deviations per joint. These parameters were selected due to their frequent use in previous research [14].

Additionally, 6 continuous waveforms (i.e., ankle angle, angular velocity, moment, power, knee angle, and hip angle) were selected as outcome measures, as they were expected to be most affected by the PAFO. Using a custom Matlab^®^ script, the quality of the continuous waveforms was visually checked by inspecting the overlaying trials and cycles for outliers or obvious marker-placement issues. Specifically, the cycles where the gait waveform exceeded by 2 standard deviations the average gait waveform for 50% of the gait cycle were excluded. For each participant, the first ‘good’ 20 gait cycles of kinematic and kinetic data were selected for further analysis. Only the participant’s most involved side, based on the clinical examination (i.e., contractures, spasticity, and strength), was considered for further analysis.

Statistical analyses were conducted both at the group level and for individual participants, to examine the differences among the 3 walking conditions. First, a total group analysis was performed based on the average gait waveform that was calculated from the 20 ‘good’ steps for each participant. Secondly, to explore patient-specific results, all separate 20 steps were utilized per participant to be used for the individual statistical analyses. For the interpretation of the individual results, the patients were grouped by barefoot gait pattern.

Shapiro–Wilk tests were used to assess the normal distribution of discrete parameters, while the normal distribution of gait cycle waveforms was evaluated using a built-in statistical parameter mapping (SPM) function (SPM—SPM1d version 0.4.7, www.spm1d.org (accessed on 10 August 2021)). Since the majority of the data were not normally distributed, medians and interquartile ranges (IQR) along with the Friedman test were used to compare the 3 walking conditions for all discrete parameters (IBM SPSS Statistics for Windows, version 27—IBM Corp., Armonk, NY, USA). Additionally, the non-parametric version of a one-way ANOVA in SPM (SnPM) with 10,000 iterations and α = 0.05 was used to highlight the differences within continuous waveforms (significant clusters or section in the gait cycle) between the walking conditions. SnPM enables hypothesis testing across entire waveforms. SnPM is freely accessible in both Python and Matlab via www.spm1d.org. It is employed to assess statistical differences between waveforms from three conditions. In SnPM, random field theory is applied to determine the critical thresholds that can be surpassed at an alpha (α) % level (here 0.05). When these thresholds are exceeded, suprathreshold clusters are formed, indicating regions within the gait cycle where statistical significance is observed. These clusters are typically reported based on their extent (or duration), range (start and end points), and *p*-values. In line with previous research, a minimal cluster duration (≥3% of the gait cycle) [22] was used to define which clusters were relevant. For the kinetics, only the clusters during the stance phase were considered.

For both statistical methods (i.e., Friedman test and SnPM ANOVA), post hoc tests (i.e., Wilcoxon signed-rank test and SnPM paired *t*-test) were conducted to identify which pairs of the 3 walking conditions showed significant differences. However, the primary focus for interpretation in the current study was on the difference between the conventional AFO and the PAFO walking conditions.

Due to the nature of the study (pilot trial), i.e., hypothesis-generating rather than hypothesis-testing, there was no correction for multiple testing for the 6 discrete parameters and the 6 continuous waveforms. Therefore, the critical *p*-value was kept at 0.05 for all statistical tests (Friedman with post hoc as well as SnPM).

## 3. Results

### 3.1. Participant Characteristics

An overview of the participants characteristics can be found in Table 1 and Table S1 in Supplementary Materials.

Eight ambulatory (GMFCS level I (50%)–III (12.5%)) children (6–17 y) with spastic uni- or bilateral (37.5%) CP were included in this study. Most participants walked with a bilateral rigid posterior leafspring (62.5%) with a rather neutral tuning angle (87–92°) and a leather inner-shoe (i.e., leather bootee; 87.5%), which was most often combined with an off-the-shelf shoe (62.5%). As part of the study design, all children wore the same type of flat shoe when combined with the PAFO (Billy Footwear^®^, Issaquah, WA, USA). In four of the eight children, the shoe was the same for the conventional AFO and PAFO condition, and all shoe types were flat. The barefoot gait patterns were classified as dropfoot (n = 2), genu recurvatum (n = 1), true equinus (n = 1), apparent equinus (n = 2), and crouch gait (n = 2).

### 3.2. Impact of PAFO on Gait for the Total Group

#### 3.2.1. Discrete Parameters

As shown in Table 2, the Friedman test revealed a significant difference (*p* = 0.034) in cadence between the 3 conditions. The post hoc test indicated a significant difference (*p* = 0.017) between the barefoot and AFO walking condition.

#### 3.2.2. Continuous Gait Waveforms

**Figure 3:**
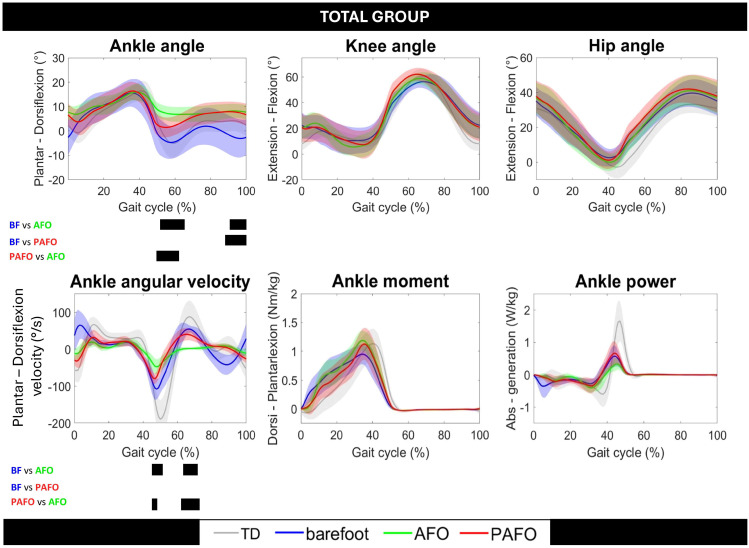
Overview of the continuous gait waveforms for the total group comparing the 3 walking conditions (i.e., barefoot = blue, with conventional ankle–foot orthosis = green, with powered ankle–foot orthosis = red). The average curve of the total group is presented in bold plotted with shaded areas of one standard deviation. The averaged data of typically developing (TD) children on the treadmill are plotted in grey as a reference. The black bars underneath the boxes indicate significant clusters within gait phases during which the SnPM{t} statistic exceeded the critical threshold in the post hoc tests.

Figure 3 entails the results of the SnPM analysis of the total group.

For the analyses of the continuous waveforms, significant differences were only found for the ankle kinematics, with one cluster around push-off (49.6–66.1%, *p* = 0.006) and one cluster at terminal swing (88.0–100%, *p* = 0.011). The post hoc test revealed significant differences between barefoot and AFO (51.6–65.4%, *p* = 0.01; 90.7–100%, *p* = 0.01), between barefoot and PAFO (88.0–100%, *p* = 0.01), and between AFO and PAFO (49.6–62.3%, *p* = 0.01). There were also significant differences between the 3 walking conditions for the waveforms of ankle angular velocity (0.0–6.0%, *p* = 0.001; 45.1–51.1%, *p* = 0.006; 62.2–73.0%, *p* = 0.001; 81.2–93.0%, *p* = 0.001). The post hoc test revealed significant differences for two clusters, between barefoot and AFO (45.1–51.5%, *p* = 0.01; 63.4–71.9%; *p* = 0.01) and between AFO and PAFO (45.1–48.3, *p* = 0.01; 62.2–73.0%, *p* = 0.01). No significant differences were found for the ankle kinetics, nor for the knee and hip.

### 3.3. Impact of PAFO on Individual Gait Patterns

**Figure 4:**
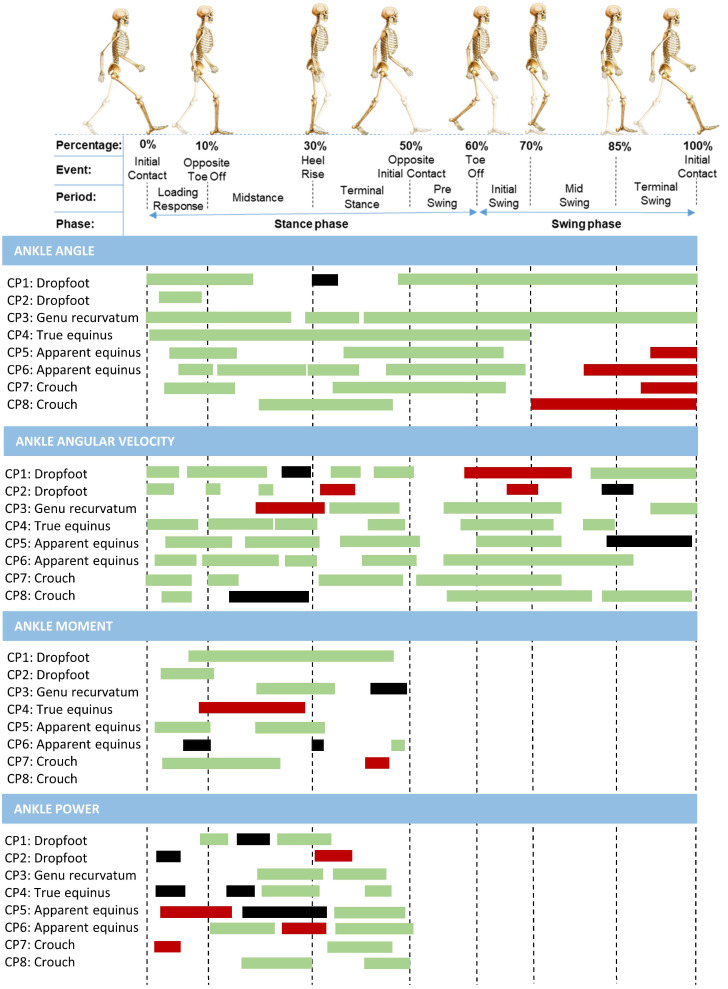
Overview of the SnPM results for each individual participant comparing the ankle kinematics (i.e., first section), angular velocity (i.e., second section), and kinetics (i.e., last two sections) between walking with their conventional ankle–foot orthosis to a powered ankle–foot orthosis. The bars indicate significant clusters within the gait cycle during which the SnPM{t} statistic exceeded the critical threshold. The colors of the bars indicate wether the gait pattern of the participant was improved (i.e., green), showed minor inconsistant changes (i.e., black), or deteriorated (i.e., red) when wearing the powered ankle–foot orthosis, taking the control data of TD children as a reference.

**Figure 5:**
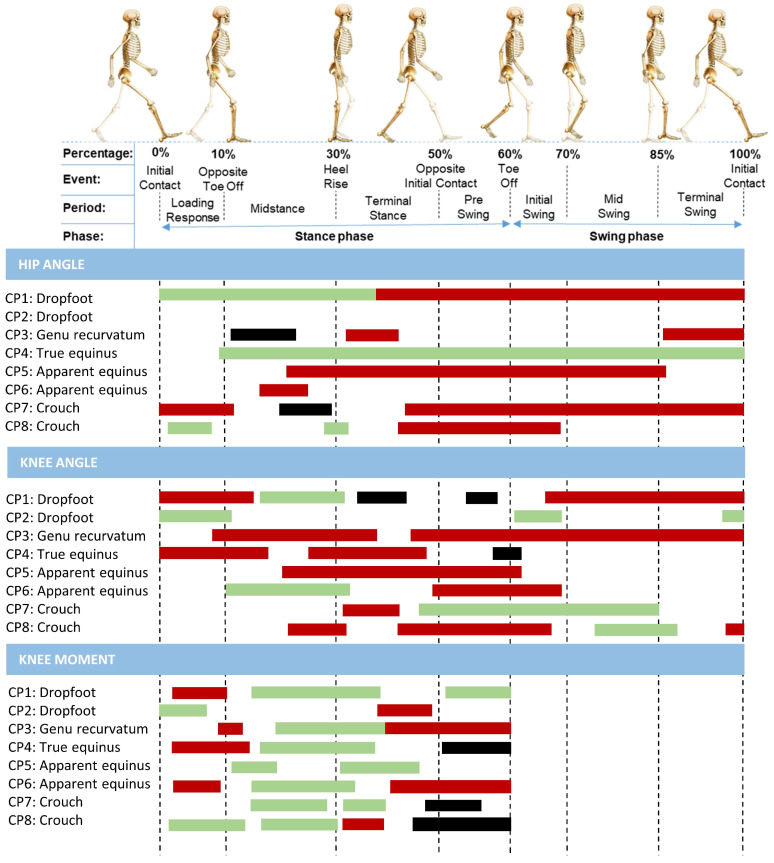
Overview of the SnPM results for each individual participant comparing the hip and knee kinematics (i.e., 1st and 2nd section) and the knee kinetics (i.e., last section) between walking with their conventional ankle–foot orthosis and a powered ankle–foot orthosis. Bars indicate significant gait phases during which the SnPM{t} statistic exceeded the critical threshold. The colors of the bars indicate wether the gait pattern of the participant was improved (i.e., green), showed minor inconsistant changes (i.e., black), or deteriorated (i.e., red) when wearing the powered ankle–foot orthosis.

The results of the SnPM analysis for the kinematics, kinetics, and angular velocity, comparing the conventional AFO with the PAFO for each participant (main focus of the current study), are described in Figure 4 and Figure 5. The horizontal bars indicate significant clusters within the gait waveform, whereby the colors of the bars specify whether the gait pattern of the participant was improved (i.e., green), showed minor inconsistent changes (i.e., black), or deteriorated (i.e., red) when wearing the PAFO in comparison to the AFO condition, taking the averaged data of the TD control group as a reference. Additionally, an overview of the kinematic and kinetic gait waveforms for the individual analyses in the three walking conditions in combination with the SnPM results can be found in Figures S1–S4 in the Supplementary Materials.

#### 3.3.1. Effects on the Ankle Joint

All participants showed improvements in ankle joint function during the stance phase while wearing the PAFO in comparison to the AFO condition. In line with the group results, nearly all participants experienced a positive effect of the PAFO during the push-off phase. The PAFO had a positive effect on the swing phase for participants with a dropfoot or genu recurvatum gait. However, the PAFO overcompensated (i.e., too much dorsiflexion) during the terminal swing phase for the children who walked with flexed gait patterns, namely apparent equinus and crouch gait.

The ankle angular velocity and ankle moment and power exhibited multiple significant clusters, which did not show clear gait-pattern-specific features. Thereby, the ankle power during the push-off phase improved across all gait patterns, except for the two dropfoot gait patterns.

Figures S1–S4 in the Supplementary Materials highlight that the ankle ROM during the push-off phase increased in the PAFO-condition in comparison to the AFO-condition for all eight children, but remained lower than the barefoot condition in five children. These individual results in the three walking conditions also highlighted the heterogeneous responses in the ankle moment and power. Yet, except for the two dropfoot patterns, the ankle angular velocity and ankle power generation during push-off always improved in the PAFO condition in comparison to the AFO as well as the barefoot condition.

#### 3.3.2. Effects on the Knee and Hip Joint

For the proximal joints, the individual SnPM analysis identified more significant clusters than what was found for the overall group. The PAFO, in comparison to the AFO-condition, negatively impacted the kinematics of the hip and knee for most participants, except for the hip kinematics of the participant who walked with a true equinus gait, who consistently demonstrated an improvement with the PAFO. Additionally, the analyses revealed that nearly all participants showed enhanced knee moments during midstance while wearing the PAFO. Figures S1–S4 in the Supplementary Materials highlight the large heterogeneity in the response of both the AFO and PAFO in comparison to barefoot walking, at the knee and the hip.

## 4. Discussion

The main goal of this pilot study was to examine whether a newly designed PAFO with patient-specific fine-tuning could improve the gait pattern of children with CP, compared to their conventional AFOs. Individual responses to PAFO assistance varied, as anticipated in this heterogeneous population. The unique value of the current study is that the PAFO settings were tuned based on the individual barefoot gait pattern. Therefore, the analyses of the results were not only performed for the total group, but also for each individual participant. For the overall group analyses, significant differences were observed in ankle kinematics and angular velocity, most obviously in the push-off phase. Yet individual analyses revealed a larger number of significant differences, highlighting the need for subgroup-specific evaluations, based on barefoot gait pattern, to capture the full range of PAFO effects.

We first expected that the PAFO would increase ankle ROM, angular velocity, and power during the push-off phase. The study findings clearly support this expectation across all gait patterns, except for the dropfoot gait. Indeed, the PAFO enabled a greater ankle ROM during push-off, specifically enhancing plantarflexion, which is typically restricted by a conventional rigid posterior leaf spring or solid AFO. Increased ankle motion and plantarflexion angular velocity contributed to enhanced ankle power during push-off in six of the eight children, addressing the greatest weakness of the conventional AFO. These results align with existing literature, suggesting that a PAFO can increase both peak plantarflexion motion and peak ankle power [8,11,24]. It should be noted that the two dropfoot cases showed unexpected largely reduced ankle power generation during push-off in the barefoot condition, in comparison to the TD children. Surprisingly, while the ankle angular velocity during push-off for these two children significantly increased while walking with the PAFO in comparison to the other walking conditions, this did not improve their impaired power generation. Dropfoot gait is often observed in children with less severe involvement and unilateral impairment [22]. These children tend to compensate for the affected side by increasing ankle power on the non-affected side. Consequently, children with unilateral involvement may not fully utilize the potential benefits of the PAFO compared to those with bilateral involvement. Future research should explore whether the PAFO provides added value for less involved unilateral children with CP and identify ways to enhance its effectiveness for this specific group.

Secondly, we expected that the PAFO would improve in the proximal joint kinematics, i.e., at the hip and knee. Indeed, previous research highlighted that well-tuned AFOs can improve the alignment of the ground reaction force (GRF) with respect to the proximal joints and may thereby address pathological motions at the knee and even the hip. Yet the current results suggested a rejection of this second a priori expectation. When compared to the conventional AFO, the PAFO did not enhance a more upright stance in the flexed gait patterns, nor did it reduce knee hyperextension in the participant with genu recurvatum gait. However, the PAFO had a positive impact on the knee moment in mid- and terminal stance in seven of the eight children, suggesting an improved GRF alignment, which is in line with what has previously been found for the conventional AFO [14]. The children probably need more time to profit from the improved kinetic conditions at the knee to achieve an overall improved gait pattern, which may explain the observed heterogeneous response to the PAFO at the knee and the hip. It can thus be concluded that, regarding the proximal joints, the PAFO did not yet have a clear added value compared to the conventional AFO. This finding could partly be explained by the fact that the conventional AFO is already able to adequately enhance the knee and hip kinematics (i.e., a more upright posture) during mid- and terminal stance by providing a stable base of support [6], leaving less room for further improvement by the PAFO. Additionally, it is important to highlight that the same actuator was used for all participants, despite the fact that for the heavier participants a more powerful actuator may be more appropriate to deliver the required assistance as needed.

While the PAFO, in comparison to the conventional AFO, generally had a positive effect on the ankle joint, it also induced compensatory adjustments in the knee and hip. These compensations may have contributed to the lack of overall improvement in overall sagittal posture during stance and the lack of a significant reduction of the overall gait index with the PAFO compared to the conventional AFO. It is important to note, however, that children with a flexed gait pattern had mild hamstring contractures and spasticity (Table S1), which can make improvements at the knee more challenging. Despite this, some children were able to achieve greater active knee extension while walking with the conventional AFO compared to the PAFO. This may be due to their greater familiarity with the conventional AFO, which could reduce the fixation (i.e., co-contraction) of the proximal joints. The improved alignment of the GRF with respect to the knee in the PAFO condition may potentially result in a correction of the flexed gait patterns when the PAFO is used within a longitudinal training program.

Due to the previously discussed improved knee moment during midstance, pointing towards a better alignment of the GRF with respect to the knee, combined with the increased ankle power during push-off, the PAFO may have reduced the energy cost of walking, which is in agreement with previous findings [14,25]. Consequently, it could be postulated that children with CP may be able to walk for longer periods at the same energy cost, which could in turn improve their participation in society. However, this needs to be further investigated in the future.

Participants appeared to tolerate the assistance well, with improvements noted after a relatively short habituation period (i.e., 30 min), in line with previous research [11]. All participants reported that the assistance was helpful and tended to rely on it after becoming accustomed to the PAFO. Some participants mentioned that walking with the PAFO felt somewhat strange initially, but they all felt safe in the laboratory setting. We used a battery-powered PAFO to ensure that our findings would be relevant to future implementation of assistive devices intended for daily use outside the laboratory. However, significant improvements are needed to make the PAFO prototype more user-friendly before advancing to this next phase. This seems feasible, since another research group recently already made substantial progress in this valorization pathway [26].

This pilot study has several limitations. First, the sample size was rather small (n = 8), primarily due to the time and budget constraints involved in creating 3D-printed, patient-tailored PAFOs and secondarily due to the time-intensive nature of the multiple-visit study design. Despite this limitation, the sample size was sufficient to demonstrate the PAFO’s working mechanism through significant differences. Moreover, the sample size of the current study is comparable to other multi-visit feasibility studies that tested newly developed ankle exoskeletons [10]. It should be noted that the cohort in this study was purposefully diverse (i.e., five different barefoot gait patterns and an age-range between 6 and 17 years). Indeed, the PAFO design was developed for the heterogenous group of ambulant children with CP, and thus needed to be tested for different gait patterns and ages. Secondly, the current focus lay solely on investigating the PAFO’s effects on kinematics and kinetics in the sagittal plane, as this was the primary plane targeted for improvement. However, gait deviations in children with CP are not limited to the sagittal plane. Therefore, future research should also explore the effects in frontal and transverse planes and on muscle activity. Thirdly, it is important to acknowledge that fatigue may have influenced the collected data. However, this unwanted effect was mitigated by incorporating rest periods between the different walking conditions. Fourthly, despite the fact that all children wore flat shoes in combination with the conventional AFO and the PAFO and four children even used the same shoes in both orthotic conditions, the type of shoe may have slightly contributed to the differences between the AFO and PAFO walking conditions. Finally, as previously mentioned, the power limit of the actuation module used in the PAFO was stretched to its limits for the heavier participants, meaning that the required assistance could not be adequately provided in terms of speed or magnitude. Yet, regardless of this non-optimal power supply for some children, the effect of the PAFO remained significant. Regarding the further development of the PAFO prototype, a detailed technical analysis may point towards potential redesigns of the assistance concept.

## 5. Conclusions

We can conclude that the PAFO improved ankle motion compared to the conventional AFO across all gait patterns. However, the effects and optimal settings of the PAFO were dependent on the specific gait pattern. This highlights the need to incorporate barefoot gait patterns into the development and testing of powered exoskeletons. The largest improvements were observed in the ankle and for patients with the most severe gait pattern, such as crouch gait. Nonetheless, the PAFO enhanced gait across all patterns based on individual needs.

## Figures and Tables

**Table 1:** Participants characteristics.

	Age (Years)	Sex	Mass (kg)	Height (cm)	Diagnosis	GMFCS	Gait Pattern *	AFO Type	AFO Tuning Angle (°)	AFO Application	Shoe Type
CP1	14–15	M	55–60	170–180	Unilateral	I	Dropfoot	Rigid PLS	89	Bi	Normal
CP2	6–7	M	25–30	120–130	Unilateral	I	Dropfoot	Hinged	87	Bi	Semi-orthopedic
CP3	16–17	F	55–60	160–170	Bilateral	II	Genu recurvatum	Rigid PLS	90	Bi	Normal
CP4	12–13	F	50–55	150–160	Unilateral	II	True equinus	Carbon fiber	90	Uni	Semi-orthopedic
CP5	14–15	M	35–40	160–170	Unilateral	I	Apparent equinus	Rigid PLS	88	Bi	Normal
CP6	10–11	F	25–30	130–140	Unilateral	I	Apparent equinus	Rigid PLS	90	Uni	Normal
CP7	10–11	M	50–55	150–160	Bilateral	II	Crouch	Neuroswing	87	Bi	Normal
CP8	12–13	F	35–40	150–160	Bilateral	III	Crouch	Rigid PLS	92	Bi	Normal

Abbreviations: CP = cerebral palsy; M = male; F = female; Uni = unilateral; Bi = bilateral; GMFCS = gross motor function classification system; AFO = ankle–foot orthosis; PLS = posterior leafspring. * Barefoot gait patterns as defined by Rodda et al. (2004) [23].

**Table 2:** Spatiotemporal and gait indices data for the different walking conditions.

	Barefoot Median (IQR)	AFO Median (IQR)	PAFO Median (IQR)
Step length (cm)	20.3 (5.2)	20.0 (5.4)	23.0 (5.4)
Cadence (steps/min) *	**79.5 (19.1)**	**65.7 (7.9)**	71.7 (14.9)
GPS (°)	9.1 (4.5)	8.9 (5.6)	8.7 (6.2)
GVS ankle (°)	7.0 (3.4)	6.6 (2.4)	5.3 (5.8)
GVS knee (°)	10.8 (4.1)	11.1 (4.7)	11.6 (6.3)
GVS hip (°)	7.3 (3.1)	6.1 (6.9)	6.3 (7.6)

* Significant difference Friedman-test (*p* = 0.034); **Bold** = significant difference post hoc test between barefoot and AFO (*p* = 0.017). Abbreviations: min = minute; AFO = ankle–foot orthosis; PAFO = powered ankle–foot orthosis; IQR = interquartile range; GPS = gait profile score; GVS = gait variable score.

## Data Availability

The data presented in this study are available on request from the corresponding author. The data are not publicly available due to privacy restrictions.

## Supplementary Materials

The following supporting information can be downloaded at: https://www.mdpi.com/article/10.3390/s24206562/s1: Table S1: Participants’ clinical examination; and Figures S1–S4: Overview of the continuous gait waveforms for each individual participant comparing the three walking conditions.

## Abbreviations

CP	Cerebral Palsy
TD	Typically Developing
AFO	Ankle–Foot Orthosis
PAFO	Powered Ankle–Foot Orthosis
ROM	Range Of Motion
GMFCS	Gross Motor Function Classification System
3DGA	Three-Dimensional Gait Analysis
GPS	Gait Profile Score
GVS	Gait Variable Score
SnPM	Statistical Non-Parameter Mapping
GRF	Ground Reaction Force
