# Supplementary material for: Evaluation of the Working Mechanism of a Newly Developed Powered Ankle–Foot Orthosis
Source: Sensors (Basel). 2024 Oct 11;24(20):6562. doi: 10.3390/s24206562 (PMC11510793; doi:10.3390/s24206562)
Supplement: Supplementary file 1 [file sensors-24-06562-s001.zip › sensors-3190391-supplementary.pdf]

Table S1. Participants clinical examination

	pROM (°)			Spasticity (MAS)			Weakness (MMT)*		Selectivity**	
	ankle DF (knee 0°)	ankle DF (knee 90°)	knee extension	gastroc-nemius	soleus	hamstrings	ankle DF	ankle PF	ankle DF	ankle PF
CP1	5	15	0	1.5	1.5	1	4	3.5	2	1.5
CP2	0	10	0	2	2	1	3	3.5	1	1.5
CP3	0	0	0	3	1.5	2	3.5	3.5	1	1
CP4	-10	-5	5	2	1.5	1.5	3	3	1	1.5
CP5	5	10	-15	1.5	1.5	1.5	2	3	0.5	1
CP6	0	5	-25	1.5	1.5	1.5	3.5	4	1.5	2
CP7	0	15	-25	2	1.5	1.5	4	4	1.5	2
CP8	10	25	-5	2	1.5	2	3	3.5	1	1

* 6-point ordinal clinical scale defined by (Daniels et al. 1986): ranging from 0 to 5, with a score of 3 indicating 'able to move against gravity' and a score of 5 referring to maximum resistance.
**5-point ordinal scale defined by (Trost et al. 2004): ranging from 0 to 2, in increments of 0.5, with a score of 2 describing a perfect selectivity
Abbreviations: pROM = passive range of motion; DF = dorsiflexion/dorsiflexors; PF = plantarflexors; MAS = Modified Ashworth Scale; MMT = manual muscle testing scale

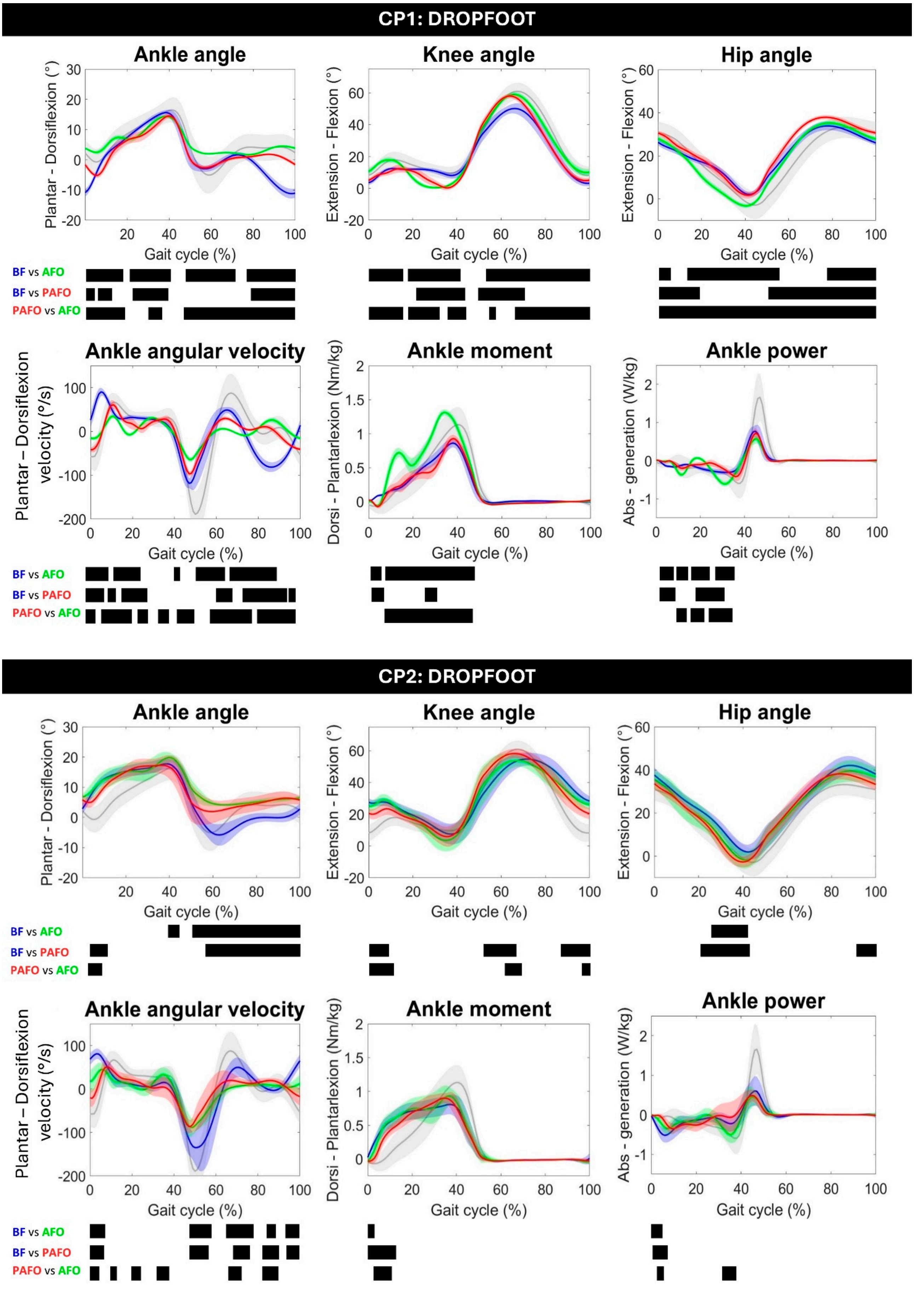

Figure S1. Overview of the continuous gait waveforms for CP1 & CP2 comparing the 3 walking conditions (i.e., barefoot = blue, with conventional ankle-foot orthosis = green, with powered ankle-foot orthosis = red). The average curve of the total group is presented in bold plotted with shaded areas of one standard deviation. The averaged data of typically developing children on the treadmill are plotted in grey as a reference. The black bars underneath the boxes indicate significant clusters within gait phases during which the SnPM{t} statistic exceeded the critical threshold in the post-hoc tests.

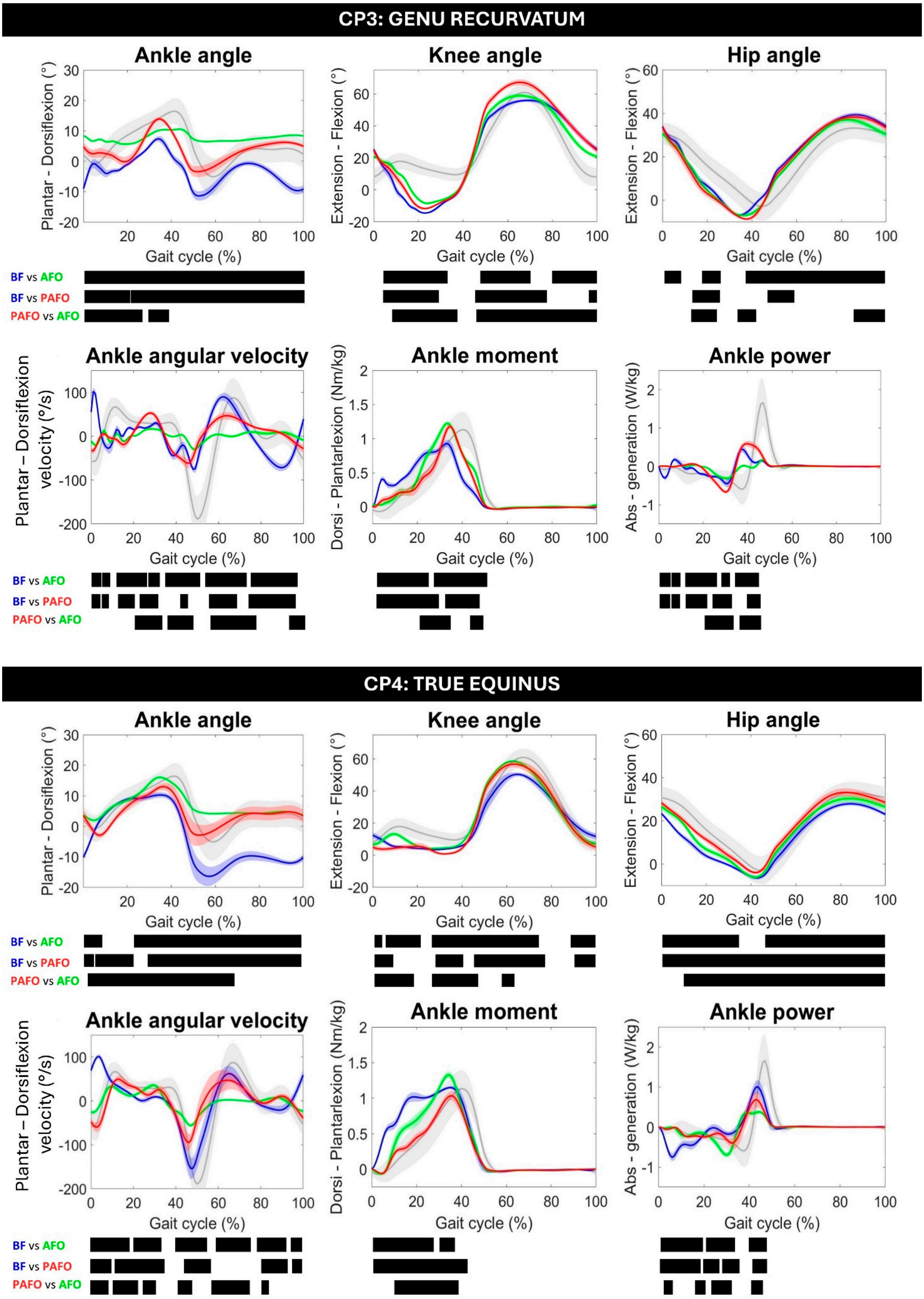

Figure S2. Overview of the continuous gait waveforms for CP2 & CP3 comparing the 3 walking conditions (i.e., barefoot = blue, with conventional ankle-foot orthosis = green, with powered ankle-foot orthosis = red). The average curve of the total group is presented in bold plotted with shaded areas of one standard deviation. The averaged data of typically developing children on the treadmill are plotted in grey as a reference. The black bars underneath the boxes indicate significant clusters within gait phases during which the SnPM{t} statistic exceeded the critical threshold in the post-hoc tests.

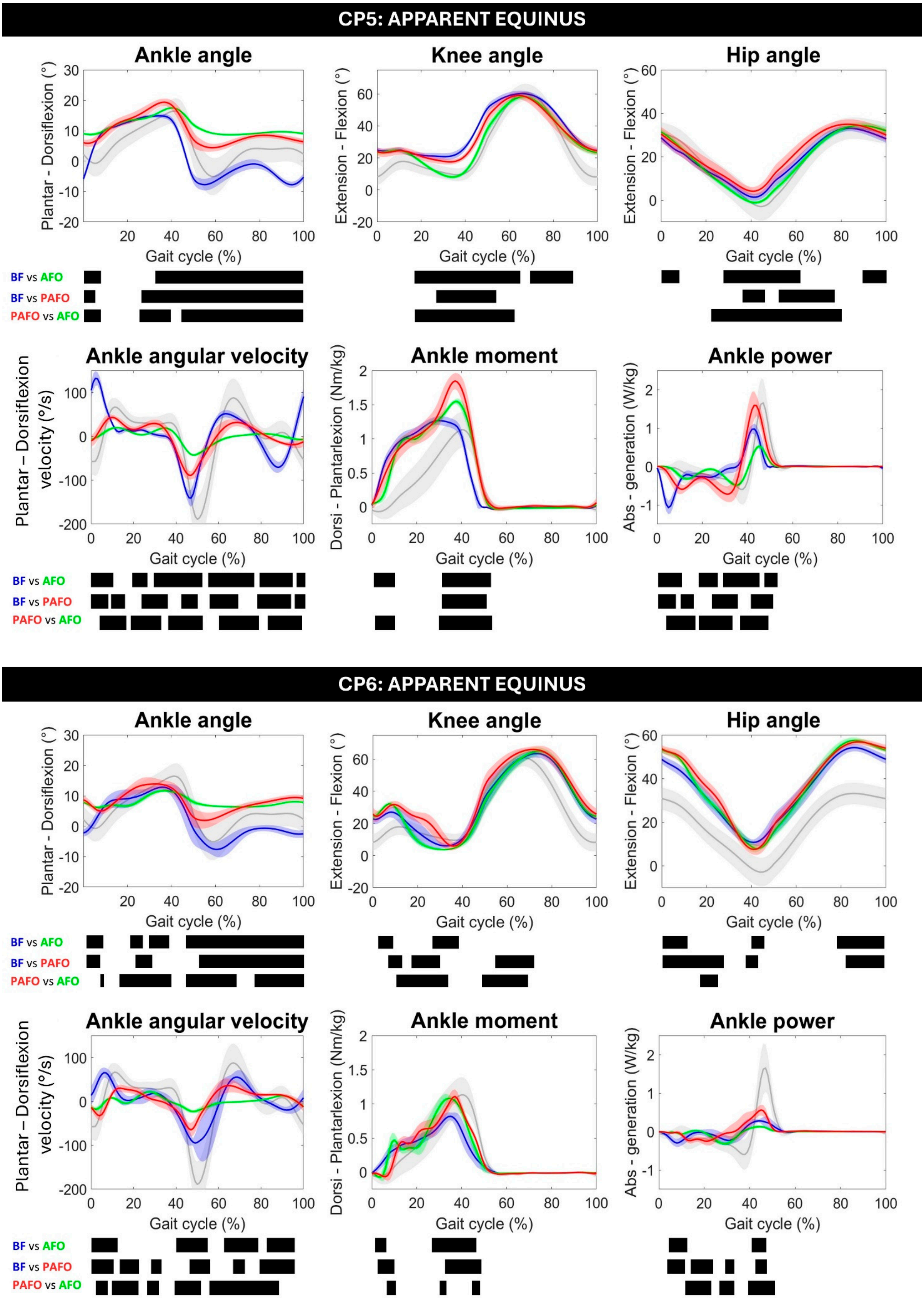

Figure S3. Overview of the continuous gait waveforms for CP5 & CP6 comparing the 3 walking conditions (i.e., barefoot = blue, with conventional ankle-foot orthosis = green, with powered ankle-foot orthosis = red). The average curve of the total group is presented in bold plotted with shaded areas of one standard deviation. The averaged data of typically developing children on the treadmill are plotted in grey as a reference. The black bars underneath the boxes indicate significant clusters within gait phases during which the SnPM{t} statistic exceeded the critical threshold in the post-hoc tests.

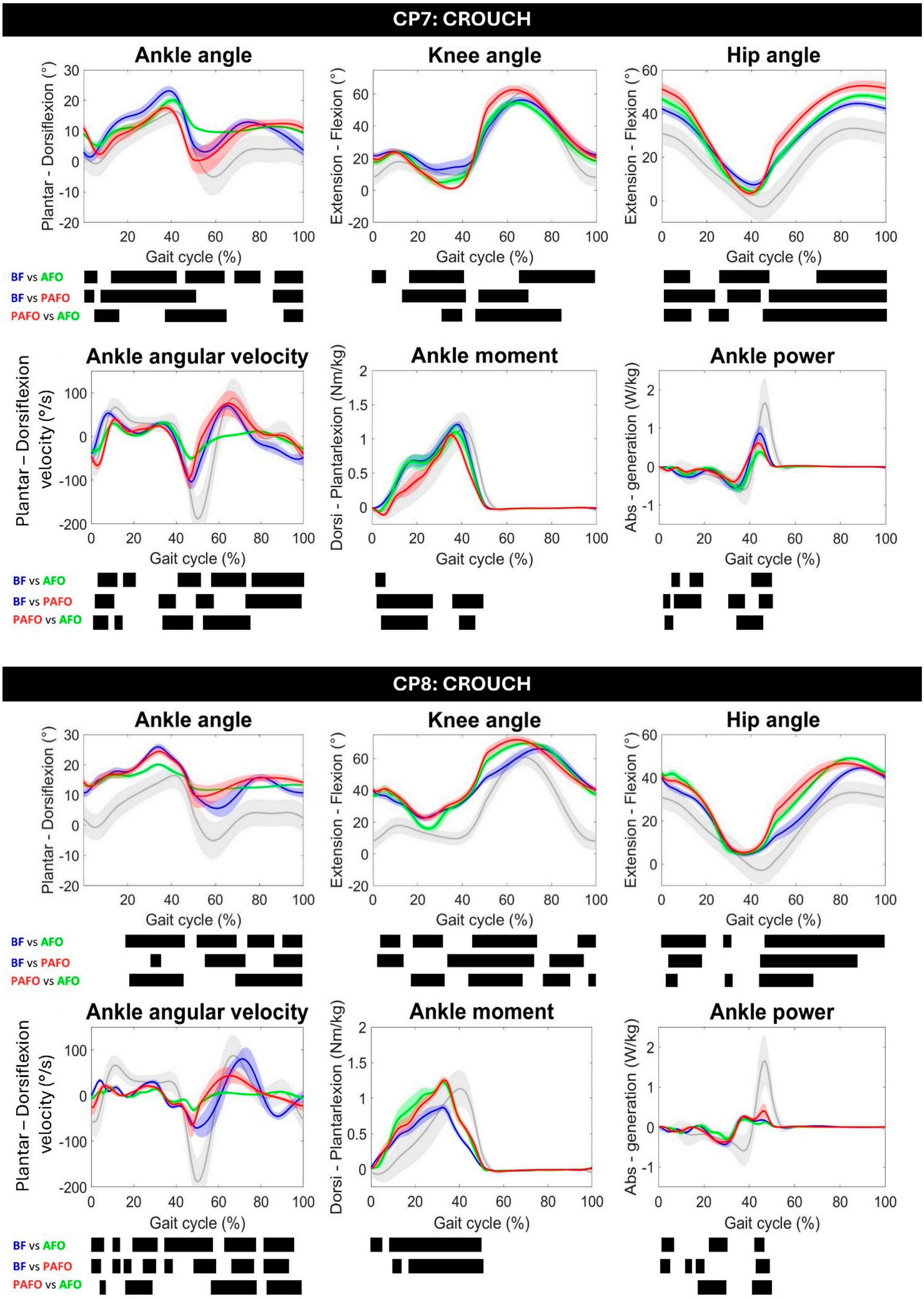

Figure S4. Overview of the continuous gait waveforms for CP7 & CP8 comparing the 3 walking conditions (i.e., barefoot = blue, with conventional ankle-foot orthosis = green, with powered ankle-foot orthosis = red). The average curve of the total group is presented in bold plotted with shaded areas of one standard deviation. The averaged data of typically developing children on the treadmill are plotted in grey as a reference. The black bars underneath the boxes indicate significant clusters within gait phases during which the SnPM{t} statistic exceeded the critical threshold in the post-hoc tests.
